# Structure and spin of the low- and high-spin states of Fe^2+^(phen)_3_ studied by x-ray scattering and emission spectroscopy

**DOI:** 10.1063/4.0000254

**Published:** 2024-10-23

**Authors:** Victoria Kabanova, Mathias Sander, Matteo Levantino, Qingyu Kong, Sophie Canton, Marius Retegan, Marco Cammarata, Philipp Lenzen, Latévi Max Daku Lawson, Michael Wulff

**Affiliations:** 1 X-ray Photon Science, Uppsala, MB 516, Sweden; 2 Paul Scherrer Institute, 5232 Villigen, Switzerland; 3 European Synchrotron Radiation Facility, CS 40220 Grenoble Cédex 9, France; 4 Synchrotron Soleil, L'Orme des Merisiers, 91190 Saint-Aubin, France; 5 Danmarks Tekniske Universitet, 2800 Lyngby, Denmark; 6 SLAC National Accelerator Laboratory, Menlo Park, California 94025, USA; 7 Department of Physical Chemistry, Science II, CH-1211 Genève 4, Switzerland

## Abstract

The structure and spin of photoexcited Fe^2+^(phen)_3_ in water are examined by x-ray scattering and x-ray emission spectroscopy with 100 ps time resolution. Excitation of the low-spin (LS) ground state (GS) to the charge transfer state ^1^MLCT^*^ leads to the formation of a high-spin (HS) state that returns to the GS in 725 ps. Density functional theory (DFT) predicts a Fe–N bond elongation in HS by 0.19 Å in agreement with the scattering data. The angle between the ligands increases by 5.4° in HS, which allows the solvent to get 0.33 Å closer to Fe in spite of the expansion of the molecule. The rise in solvent temperature from the return of photoproducts to the GS is dominated by the formation dynamics of HS, ^1^MLCT^*^ → HS, which is followed by a smaller rise from the HS → GS transition. The latter agrees with the 0.61 eV energy gap E(HS)−E(LS) calculated by DFT. However, the temperature rise from the ^1^MLCT → HS transition is greater than expected, by a factor of 2.1, which is explained by the re-excitation of nascent HS^*^ by the 1.2 ps pump pulse. This hypothesis is supported by optical spectroscopy measurements showing that the 1.2 ps long pump pulse activates the HS^*^ → ^5^MLCT^*^ channel, which is followed by the ultrafast return to HS^*^ via intersystem crossing. Finally, the spins of the photoproducts are monitored by the K_β_ emission and the spectra confirm that the spins of LS and HS states are 0 and 2, respectively.

## I. INTRODUCTION

Electron transfer in transition metal complexes can convert photon energy into electric or chemical energy which has found applications in solar cells and photocatalysis.^1^ In photosynthesis, the transfer of an electron from a metal to a ligand is responsible for the creation of a chemical potential that activates ATP, the energy carrier in biological systems.^2^ In photocatalysis, the light driven splitting of water into dioxygen and dihydrogen, catalyzed by a photoactivated Ru(II) complex, is an example of the strive toward cleaner and renewable energy.^3^

In transition metal complexes with open 3d, 4d, or 5d shells, a d electron is transferred upon photon absorption to a ligand orbital, and this state is called the metal-to-ligand charge transfer (MLCT) state. The process is reversible and the electron returns to the d orbital of the metal. In 4d and 5d complexes, the LMCT lifetime is long, from nanoseconds to microseconds. In 3d complexes it is of the order of sub-picoseconds in most cases. In 4d and 5d complexes, the outer electron density is predominantly 4d or 5d in character and the ligand bonding is stronger than for 3d complexes. The ligand field splitting of 4d or 5d orbitals is therefore greater than for 3d complexes. In fact, the lowest excited state for 4d and 5d complexes is the LMCT state whereas for 3d complexes it is a metal centered (MC) ligand field state. In the decay of the MLCT state in 3d complexes, the electron returns to the ground state (GS) via the MC state which greatly shortens the LMCT lifetime compared with the equivalent 4d and 5d systems.^4^ In the 4d and 5d complexes Ru^2+^(bpy)_3_ and Os^2+^(bpy)_3_, the MLCT lifetimes are 900 (Ref. 5) and 25 ns (Ref. 6), respectively, whereas it is 0.96 ps for Fe^2+^(bpy)_3_ .^7^ The short MLCT lifetime of 3d complexes makes them unsuitable for light conversion since the charge separated state does not have time to interact with the environment. Since 4d and 5d metals are rare and expensive, it is important to synthetize ligands for 3d systems with the strongest possible ligand field. The ligand field strength depends on the electronegativity of the ligand atoms in the metal–ligand bonds: O and halogens produce weak fields, N intermediate fields, and C and P strong fields. In the carbene complex Fe^3+^(btz)_3_(PF_6_)_2_, the strong Fe–C *σ* bonds generate a high electron density on Fe^3+^ and the MC states are inaccessible in the decay process. The MLCT state decays directly to the GS by photon emission with a long 528 ps lifetime.^8^ Another example is the Fe^3+^(phtmeimb)_2_ complex, which has a record MLCT lifetime of 2 ns.^9^

In this work we present a time resolved study of the change in structure and spin of the HS state of Fe^2+^(phen)_3_ (phen = 1,10-phenanthroline) in water using wide-angle x-ray scattering (TRWAXS) and x-ray emission spectroscopy (TRXES). The aim is to measure the change in the average Fe–N bond length to the ligands, the relative ligand angles, the spin states of Fe as well as the cage structure and hydrodynamics of the solvent. The work was performed by laser/x-ray pump probe using 100 ps x-ray pulses from the European Synchrotron Radiation Facility (ESRF).^10,11^ The experimental details are given in Sec. II and in the supplementary material.^35^

**FIG. 1.**
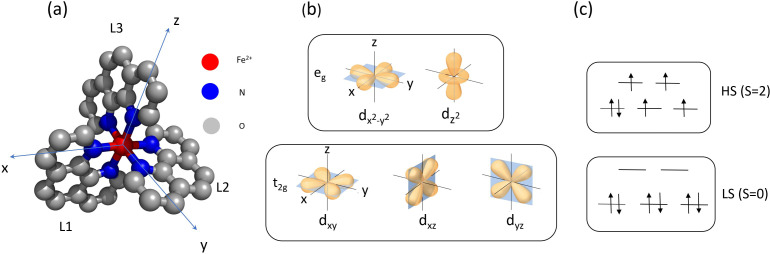
(a) Structure of Fe^2+^(phen)_3_ with Fe^2+^ in red, N in blue, and C in gray. H atoms are omitted. (b) In an octahedral ligand field, the five basic 3d wavefunctions are split into t_2g_ and e_g_ energy levels. (c) LS and HS configurations for the six 3d electrons in Fe^2+^.

Fe^2+^(phen)_3_ has the stoichiometry Fe^2+^(N_2_C_12_H_8_)_3_ with 67 atoms per molecule. Phenanthroline, a medium strong ligand, has two Fe–N bonds per ligand in Fe^2+^(phen)_3_. The LS and HS structures were calculated by density functional theory (DFT) using the B3LYP function and the 6-311++G(3df,3pd) basis set. The effect of the solvent was included using the polarizable continuum model (CPCM) with Fe^2+^(phen)_3_ placed in a cavity in the solvent field. The LS structure was assumed to have D_3_ symmetry in the calculation. The LS calculation predicts six equal Fe–N bond lengths of 1.99 Å and relative ligand angles of 88.8°. The HS state was assumed to have C_2_ symmetry. The Fe–N bonds and the relative ligand angles were calculated to be 2.18 Å and 94.2° on average. The LS structure is shown in Fig. 1(a). The atomic positions and structural parameters are listed in Appendix 1 in the supplementary material.^35^

The 3d orbitals in the ligand field states t_2g_ and e_g_ are shown in Fig. 1(b) and the LS and HS configurations are shown in Fig. 1(c).

In the ground state t_2g_, the lopes of the 3d_xy_, 3d_xz_, and 3d_yz_ wavefunctions are pointing along the diagonals in the (x, y), (y, z), and (z, x) planes towards the midpoints of the N-N lines of the ligands where the repulsive energy is lowest. In e_g_ the d_x2-y2_ and d_z2_ lopes are pointing along the x, y, and z axes in the direction of the N atoms. The repulsive energy is higher and the Fe–N bonds are longer.

The electron configuration of Fe^2+^ is [Ar]3d^6^. The 3d orbitals are tenfold degenerate and can accommodate five up-spins and five down-spins. The six 3d electrons in Fe^2+^are distributed in t_2g_ and e_g_ according to Hund's rule: maximize the total spin at the lowest possible total energy. If the ligand field splitting is greater than the spin-pairing energy in an orbital, the case for Fe^2+^(phen)_3_, the three orbitals in t_2g_ are filled with three up- and three down-spins. The total spin is S = 0. For the next 7–10 electrons in the following 3d metals, e_g_ is filled by maximizing the spin. For ten 3d electrons, the total spin S is  0. If the ligand field splitting is smaller than the spin-pairing energy, the first five electrons have parallel spins in t_2g_ and e_g_. The next five electrons are then put in t_2g_ and e_g_ with antiparallel spins.

As mentioned, phenanthroline is a medium strong ligand and the GS of Fe^2+^ (phen)_3_ has six 3d electrons in t_2g_ with antiparallel spins and S = 0. When the e_g_ orbitals are populated in HS at the end of the photocycle, e_g_ receives the recombining electron which triggers the transfer of one t_2g_ electron to e_g_ with a change in spin. The two t_2g_ electrons have parallel spins and the total spin S is  2.

The photocycle of Fe^2+^(phen)_3_ in acetonitrile was studied by ultrafast optical spectroscopy by Tribollet *et al.*^12^ and Gallé *et al.*,^13^ by time resolved EXAFS in aqueous solution by Nozawa *et al.*,^14^ and by XANES by Wang *et al.*^15^ The Fe–N bond lengths derived from the EXAFS work are 1.98(1) and 2.15(1) Å for the LS and HS states, respectively. The HS lifetime was reported to be 690 and 685 ps in the EXAFS and XANES works, respectively. For comparison, the HS lifetimes of the sister complexes Fe^2+^(terpy)_2_ and Fe^2+^(bpy)_2_ in aqueous solutions are 2.61 ns (Ref. 34) and 0.665 ns (Ref. 7), respectively, and the Fe^2+^(btbip)_2_ complex has a HS lifetime in acetonenitrile of 0.23 ns (Ref. 16).

**FIG. 2.**
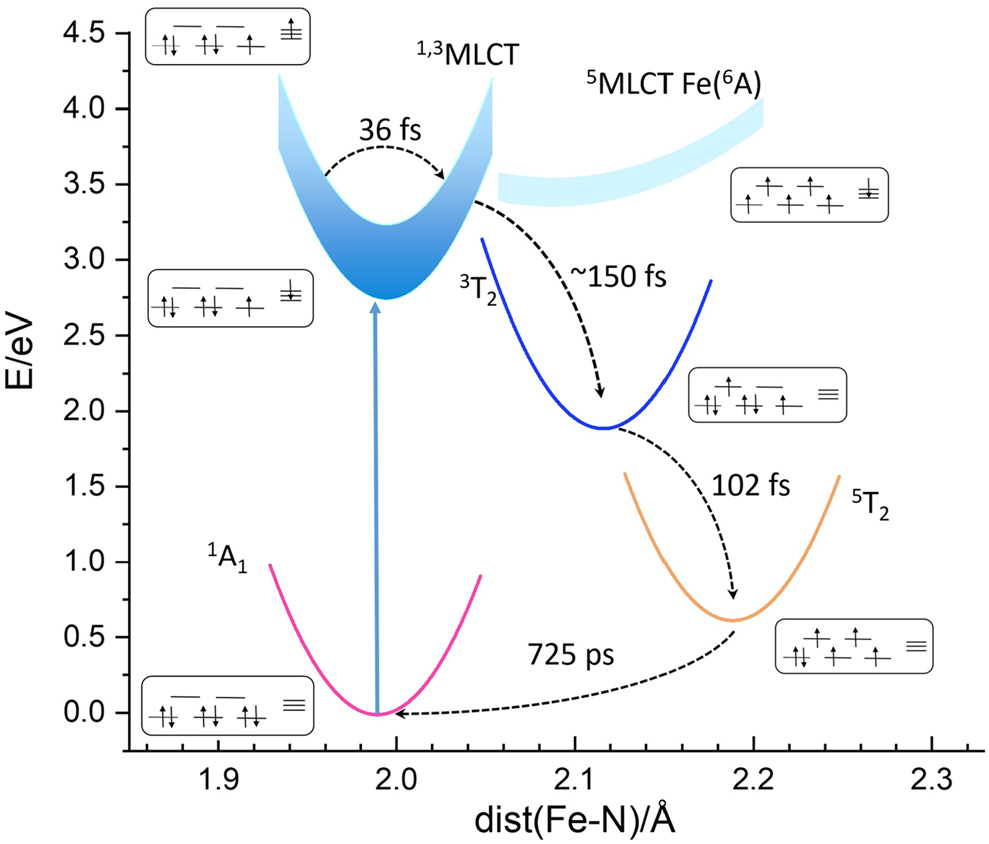
(a) Calculated energy curves and spin states for Fe^2+^(phen)_3_ vs the Fe–N bond distance. The data are adopted from Sousa *et al.*^17^

The Fe^2+^(phen)_3_ wavefunctions, transition rates, and energy curves were calculated in acetonitrile by Sousa *et al.*^17^ and the energy curves and spin states are summarized in Fig. 2. The photocycle proceeds as follows. When Fe^2+^(phen)_3_ is excited from the GS ^1^A_1_, a 3d electron in t_2g_ moves to one of the ligands to form the ^1^MLCT state. The ^1^MLCT transits in 36 fs to the triplet ^3^MLCT that in turn decays to the MC triplet ^3^T_1_ in 150 fs. ^3^T_1_ decays in turn to ^5^T_2_ in 102 fs by intersystem crossing (ISC). The ^5^T_2_ state is reached in ∼300 fs. ^5^T_2_ is initially in an excited vibrational state that relaxes to the bottom of the potential in a few picoseconds via vibrational cooling by the solvent. The final step ^5^T_2_ → ^1^A_1_ proceeds via ISC in 1.1 ns in acetonitrile. Note that ^3^T_1_ and ^5^T_2_ have one and two antibonding electrons in e_g_, respectively, which explains the progressive elongation of the Fe–N bonds.

The HS(S = 2) state was first observed by Tribollet *et al.*^12^ Their Fe^2+^(phen)_3_/acetonitrile solution was excited by 30 ps pulses at 540 nm and probed by 30 ps pulses of white light. They observed a transient absorption feature around 340 nm with a 1.1 ns lifetime that they assigned to the HS state ^5^T_2_. The absorbing feature was assigned to the ^5^T_2_ → ^5^MLCT excitation.

## II. EXPERIMENTAL DETAILS

TRWAXS is used in this work to monitor the structural changes in the LS to HS transition, i.e. the change in Fe–N bond lengths, the relative ligand angles, the effective radius of the solvent cage, and the change in the solvent temperature. As 1D scattering data cannot be inverted to electron density directly, the measured scattering curves were compared with the predictions from combined DFT and MD simulations. The change in the spin state of Fe^2+^ was probed by TRXES from the K_β_ emission line. The experiments were performed on beamline ID09 at the European Synchrotron Radiation Facility (ESRF) and the time resolution was obtained by pump and probe. A 10 mM aqueous solution of Fe^2+^(phen)_3_ was circulated in a liquid jet, excited by 1.2 ps pulses at 400 nm and probed by 100 ps x-ray pulses from an undulator. In the TRWAXS study, 18 keV x-ray pulses were produced by a 2.1% bandwidth (BW) Ru multilayer (ML) to maximize the intensity while keeping the beam quasi monochromatic. The intensity of the ML beam was 4 × 10^8^ ph/pulse and the pump & probe pulses were repeated at 986.3 Hz. In the TRXES experiment, the 5% BW pink beam from the fundamental of the U17 undulator at 15 keV was used to generate 1s core holes on the Fe K-edge. The intensity of the pink beam was 2 × 10^9^ ph/pulse. With the 100 ps time resolution from the x-ray pulse length, the HS state is the only structure resolved in the present experiment. The setup and the experimental parameters are described in more detail in the supplementary material.^35^

## III. SCATTERING RESULTS

The structural information from TRWAXS data is obtained by fitting candidate structures from DFT/MD simulations against the laser induced change in the scattering ΔS(q, t). The LS and HS structures were calculated by density functional theory (DFT) as mentioned earlier. From the DFT atomic positions, the scattering from randomly oriented Fe^2+^(phen)_3_ molecules was calculated by the Debye function as follows:^18^

$$S(q)=\sum_{n,m}\;f_{n}(q)\;f_{m}(q)\;\frac{\text{sin}\left(q\;r_{nm}\right)}{\left(q\;r_{nm}\right)},$$ (1)

where r_mn_ is the distance between atom pairs and f_n_(q) is the atomic form factor of atom n. The forward intensity S(q = 0) is the coherent intensity from all the electrons in the molecule (Σ Z_i_)^2^, with the sum extending over all atoms in the molecule and Z_i_ being the number of electrons in atom i. Fe^2+^(phen)_3_ has 306 electrons per molecule, S(q = 0) = (306 e.u.)^2^ = 93 636 (e.u.)^2^ where e.u. is the *electron unit* defined by the atomic form factor.^18^ As the stoichiometry of the LS and HS structures does not change, the change in the forward scattering ΔS(q = 0) is zero. In contrast, if a molecule dissociates or associates, ΔS(q = 0) is negative or positive, respectively.

**FIG. 3.**
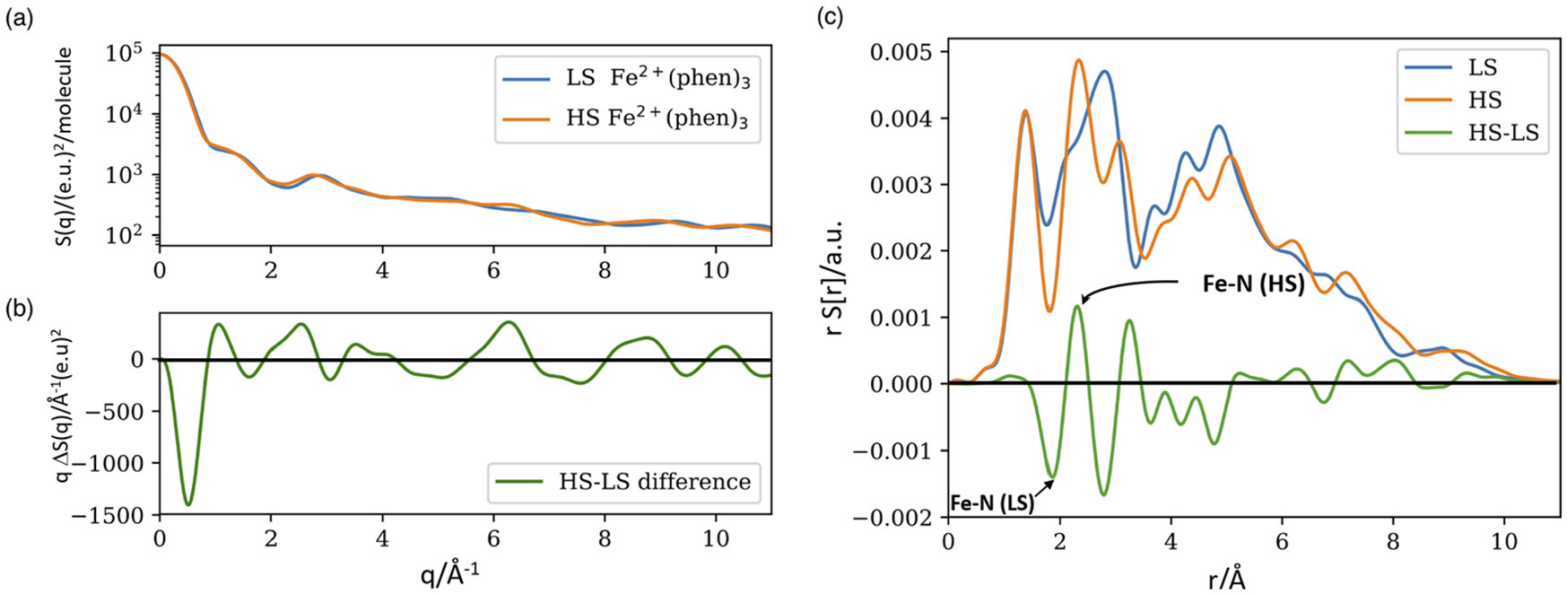
Scattering functions for Fe^2+^(phen)_3_. (a) The total scattering for the DFT structures of the LS and HS states. (b) Difference scattering qΔS(q) (HS-LS). The negative part between 0 and 1 Å^−1^ is the signature of the Fe–N bond extension in the HS state. (c) Fourier transforms rS[r] and rΔS[r]. The assignments of the LS and HS contributions are indicated on the HS-LS curve.

The scattering functions S(q) for the LS and HS structures are shown in Fig. 3(a) and qΔS(q) are shown in Fig. 3(b). q multiplication is used to amplify the high-q spectrum, which is particularly sensitive to intra molecular structural changes. Note that qΔS(q) has 12 nodal points in the range 0 < q < 10.7 Å^−1^, which is a very stringent constraint for the comparison of calculated and measured scattering curves.

The change in the radial electron density for an average excited atom is obtained from the Fourier transform,^19^

$$\Delta S[r,t]=\frac{1}{2\pi^{2}\;r}\;\int_{0}^{\infty }dq\;{\left[\sum_{\mu \neq \nu }f_{\mu }\;(q)\;f_{\nu }\;(q)\right]}^{-1}q\;\Delta S(q,\;t)\text{sin}(qr).$$ (2)

Δ*S*[r,t] is the form factor biased change in the radial electron density which is biased by the high-Z atom in the molecule. The square brackets in S[r] is used to show that it is the Fourier transform of S(q). The rS[r] and rΔS[r] functions are shown in Fig. 3(c). The green curve rΔS[r] has a negative peak at 1.87 Å and a positive at 2.29 Å which is the signature of the Fe–N bond elongation in the HS state. There is a small difference between the r values of the peak and valley positions compared with the DFT values of 1.99 and 2.18 Å which is due to the interference of positive and negative peaks in the difference curve.

**FIG. 4.**
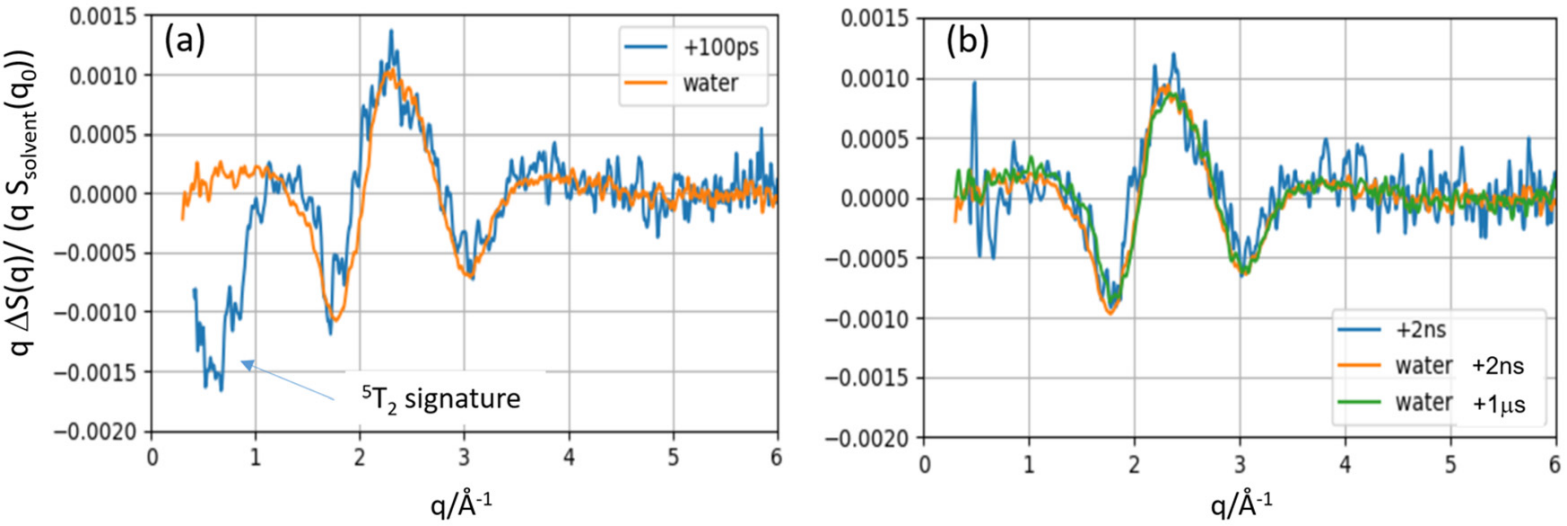
qΔS(q,t) scattering curves for time delays of 100 ps and 2 ns. (a) The blue curve probes the total difference scattering from the solute, cage, and solvent. The orange curve is the solvent part alone. (b) After 2 ns, the blue and orange curves coincide as the HS population has returned to the GS; the residual orange signal is purely thermal.

The measured curves ΔS(q, t) have two principal contributions: one from the change in the solute/cage structure and one from the change in the solvent temperature from the relaxation of the photoproducts. The measured qΔS(q, t) curves for 100 ps and 2 ns are shown in Fig. 4. The blue curve in Fig. 4(a) is the total change in the solute/cage/solvent scattering (HS-LS). The solvent part is produced by the adiabatic temperature rise in the solvent after 100 ps. The orange curve is the solvent contribution that was measured in the separate dye/water experiment described in the supplementary material.^35^ The difference between the curves is the intrinsic change in the solute and solute cage structure. The negative part between 0.5 and 1.0 Å^−1^ in the blue curve is the signature of the Fe-N bond extension in HS. The rise in the solvent temperature after 100 ps is produced by the ultrafast cascade ^1^MLCT → ^3^MLCT → ^3^T_2_ → ^5^T_2_ shown in Fig. 2. For the 2 ns curves in Fig. 4(b), the total solute/cage/solvent curve in blue coincides with the dye/water curve: the HS state has returned to the GS and the residual signal is from the adiabatic temperature rise of the solvent. Note that at fast timescales t < 10 ns, the solvent temperature rises monotonically and that the rise accumulates all relaxation processes in the photocycle, including the ultrafast ones that are not resolved structurally with 100 ps time resolution.

The normalization of the intensity is done by scaling the non-excited total scattering to 1.0 at q_0_ = 6.7 Å^−1^ and using that calibration for the difference scattering. The total scattering from a 10 mM “unit-cell” solution of Fe^2+^(phen)_3_ in water, elastic plus Compton scattering, is 13.3 (e.u.)^2^/unit.^20,21^

## IV. SCATTERING MODEL

The experimental data were analyzed with the following terms:

$$\Delta S(q,t)=\gamma (t)({\Delta S}_{\text{solute}}(q)+{\Delta S}_{\text{cage}}(q))+\Delta S_{\text{solvent}}(q,t),$$ (3)

where *γ*(t) is the HS population vs time. The solute term is the Debye scattering from the DFT structures and the cage term is calculated from the atom–atom distribution functions g_ij_(r) from the MD simulation,^27^

$$\Delta S_{\text{cage}}(q)=\sum_{n}\sum_{m\neq n}(\frac{N_{n}\;N_{m}\;}{V}\;f_{n}(q)\;f_{m}(q)\int_{0}^{\infty }\Delta g_{nm}(r)\frac{\text{sin}\left(\;q\;\cdot r\;\right)}{\;q\;\cdot r}4\pi r^{2}\;dr),$$ (4)

where (n,m) represents all solute–solvent pairs. The solvent term has two contributions,^22–24^

$$\Delta S_{\text{solvent}}(q,t)=\Delta T(t)\frac{\partial S(q)}{\partial T}{|}_{\rho }+\Delta \rho (t)\frac{\partial S(q)}{\partial \rho }{|}_{T}.$$ (5)

The second term in Eq. (5) is not needed here since the timescale in the experiment is limited to 0–2 ns where the solvent density is constant.

**FIG. 5.**
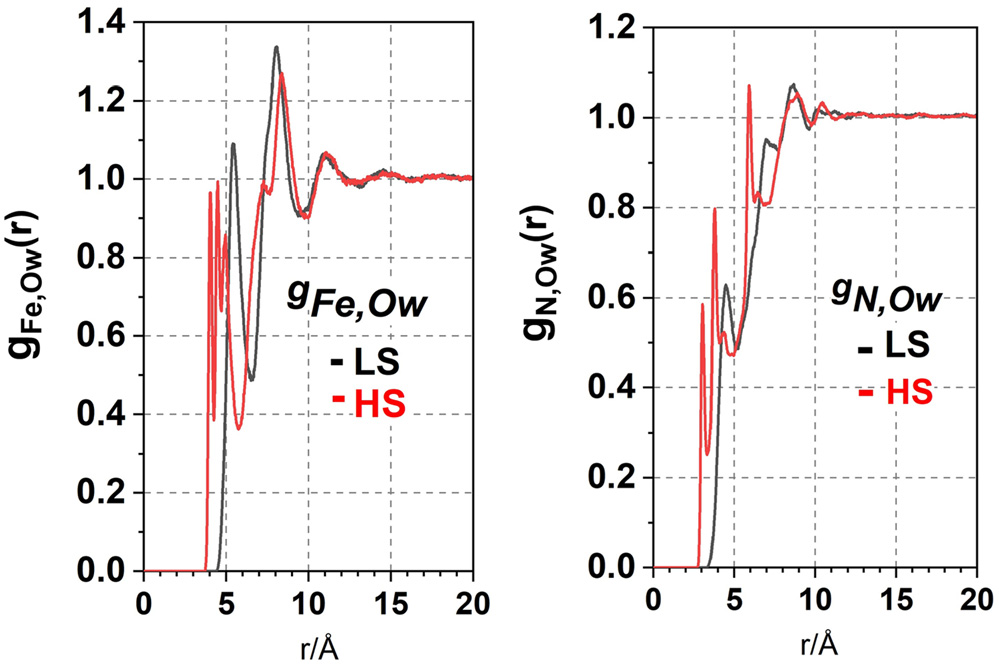
(a) The MD calculated distribution function g(Fe, O_w_) showing the position of oxygen in water in the LS and HS states. The oxygen in water moves about 1.0 Å closer to Fe in HS in spite of the 0.19 Å Fe–N bond elongation in HS. (b) The g(N, O_w_) function shows as well that O_w_ moves closer to N in HS.

The MD simulations of the solvent structure around LS and HS states were performed by MOLDY employing the Lennard–Jones potentials from the TIP4P/2005 model of water.^25^ In the simulation, the LS and HS structures of Fe^2+^(phen)3 were placed in a (49.6 Å)^3^ box with 4096 water molecules. The atom–atom distribution function g_Fe,Ow_(r), where O_W_ is oxygen in water, is a measure of the effective cage radius. The g_Fe,Ow_(r) and g_N,Ow_(r) functions are shown in Fig. 5.

**FIG. 6.**
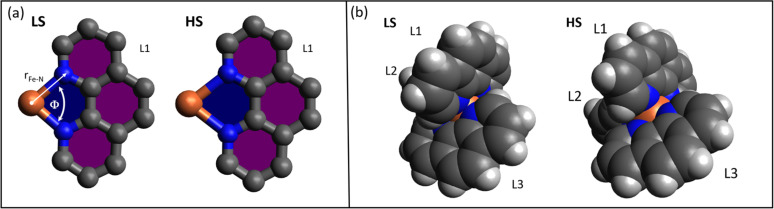
(a) Position and angle of the ligand L_1_ in the LS and HS states from the DFT calculations. Fe is shown in orange, N in blue, and C in gray. For L_1_ the Fe–N bond expands from 1.99 to 2.18 Å in LS and HS respectively. The N–Fe–N angle ϕ gets smaller in the expansion, from 82.6° to 76.93°. (b) The angles between adjacent ligand planes increase by 5.4° on average in HS. The solvent moves closer to Fe in HS as illustrated by the orange van der Waals sphere.

The first peak in the LS function g_Fe,Ow_(r) at 5.5 Å shifts to ∼4.5 Å in HS. The latter has three sub-peaks separated by about 0.4 Å. The splitting is caused by the distortion of the HS structure, in particular the slightly different relative ligand angles in HS that vary from 90.4° to 96.0°. When the Fe^2+^(phen)_3_ structure is rendered with 3D van der Waals spheres, the increased solvent accessibility to Fe in HS is evident, see Fig. 6. This accounts for the counterintuitive fact that water moves closer to Fe in HS in spite of the Fe–N elongation.

A splitting of the g_Fe,Ow_(r) function in HS was also observed in MD simulations of Fe^2+^(bpy)_3_ in water by the Hauser group^26^ although the effect is less pronounced.

**FIG. 7.**
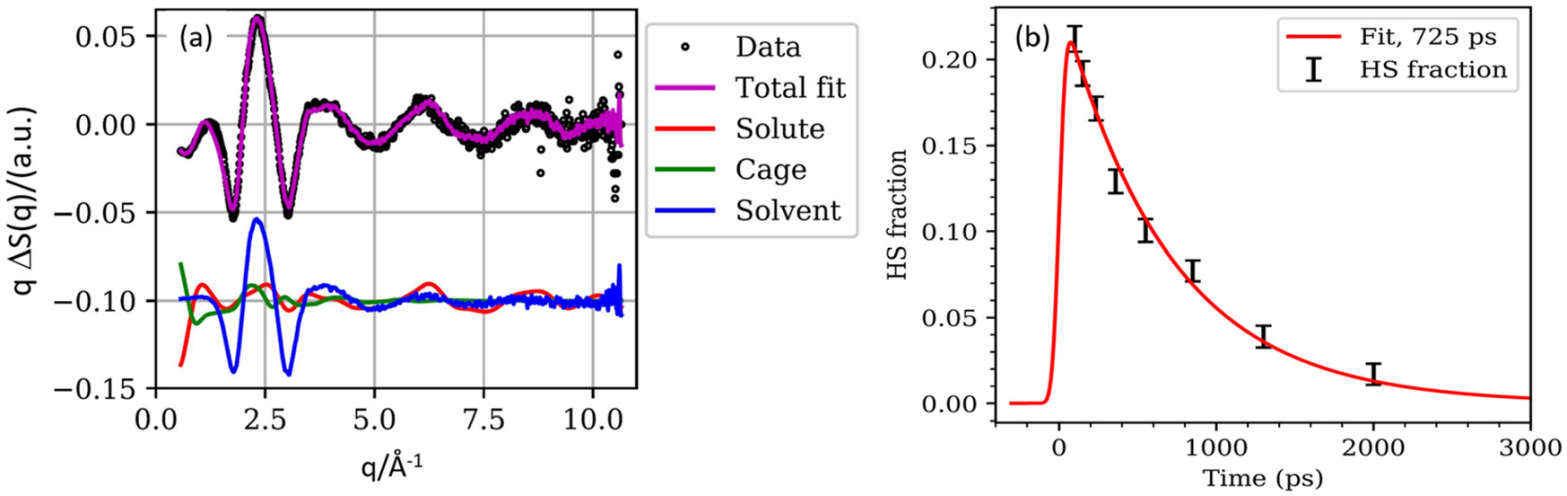
(a) The total scattering qΔS(q,100 ps) is shown with dots and the solute, cage, and solvent contributions are shown in red, green, and blue, respectively. The purple curve is the sum of components. The amplitude of the cage term is reduced to 33% of the MD value to fit the data optimally. The upturn in the cage term at low q is the signature of the cage contraction in HS. (b) The HS population inferred from the solute amplitude in Fig. 7(a) as a function of time. The red line is the fit to the data with an exponential 725 ps time constant and 100 ps x-ray pulse (FWHM).

The solute, cage, and solvent contributions to the total scattering are shown in Fig. 7(a) together with the time dependence of the HS population γ(t) shown in Fig. 7(b). The red curve in Fig. 7(b) is a Gaussian–exponential fit to the HS population with a 725 ps time decay and a 100 ps probe pulse (FWHM). The onset and decay are fitted to the convolution of an exponential decay and a Gaussian x-ray pulse as,

$$S(\tau )=\frac{1}{\sqrt{2\pi \;\sigma }}\;\int_{0}^{\infty }dt\;\text{exp}(-\frac{t}{\tau_{R}})\;\text{exp}(-\frac{{\left(t-\tau \right)}^{2}}{2\sigma^{2}})=\frac{1}{2}e\text{xp}(\;\frac{\sigma^{2}-2\tau \;\tau_{R}}{2\tau_{R}^{2}})\;\{1-\text{erf}(\frac{\sigma^{2}-\tau \;\tau_{R}}{\sqrt{2}\;\sigma \;\tau_{R}})\},$$ (6)

where *τ* is the laser/x-ray delay, *τ*_R_ is the HS time constant, and *σ* the duration of the x-ray pulse. The extrapolated fraction of HS molecules at time zero is 24.3% of the 10 mM solution.

To optimize the fit of the experimental data to the DFT/MD model, the amplitude of ΔS_cage_(q) is reduced to 33% of the MD calculated value. MD tends to overestimate the cage amplitude which is due to the fact that MD simulations are using rigid solute structures that allows the solvent to get closer to the solute.

The amplitude of the total scattering curves ΔS(q,t) decreases exponentially with a 725 ps time constant. The shape of the spectra vs. time does not change which is consistent with the fact the HS → LS transition is ultrafast for single molecules. By contrast, the amplitude decreases vs time from the decay of the population of the HS state.

 Note that the cage signal is greatest between 0-2.5 Å^−1.^ and that the positive part signals the contraction of the cage. In the low q range 0.3 – 0.8 Å^−1^, a small shift dr in the atom–atom distribution leads to a proportional change in the *amplitude* of ΔS(q) as shown in the supplementary material.^35^ In that approximation, the cage radius contracts by 0.33 Å in response to the metal–ligand elongation in the HS state.

The cage dynamics for Fe^2+^(bpy)_3_ in water was studied by TRWAXS by Khakhulin *et al.*^27^ and Haldrup *et al.*^28^ In Khakhulin's work, the solute and cage were calculated by *ab initio* MD (AIMD), which is a more realistic account of the solute/cage structure in water. In this complex the Fe–N bond extends from 1.99 Å (LS) to 2.18 Å(HS) and the AIMD calculation showed that the first peak in g(Fe, O_w_)moves from 5.29 Å in LS to 5.51 Å in HS corresponding to a 0.22 Å expansion of the cage. The difference in the cage dynamics in Fe^2+^(phen)_3_ and Fe^2+^(bpy)_3_ is ascribed to the difference in the geometry of the ligands and the different structural distortions in the HS states.

## V. SOLVENT TEMPERATURE

As the temperature term dominates the dS(q,t) curves it is essential to have accurate solvent differentials to isolate the solute/cage contribution from the thermal background. This was done in a separate water/dye experiment performed with the same experimental conditions as the main experiment. The dye experiment is described in the supplementary material.^35^

**FIG. 8.**
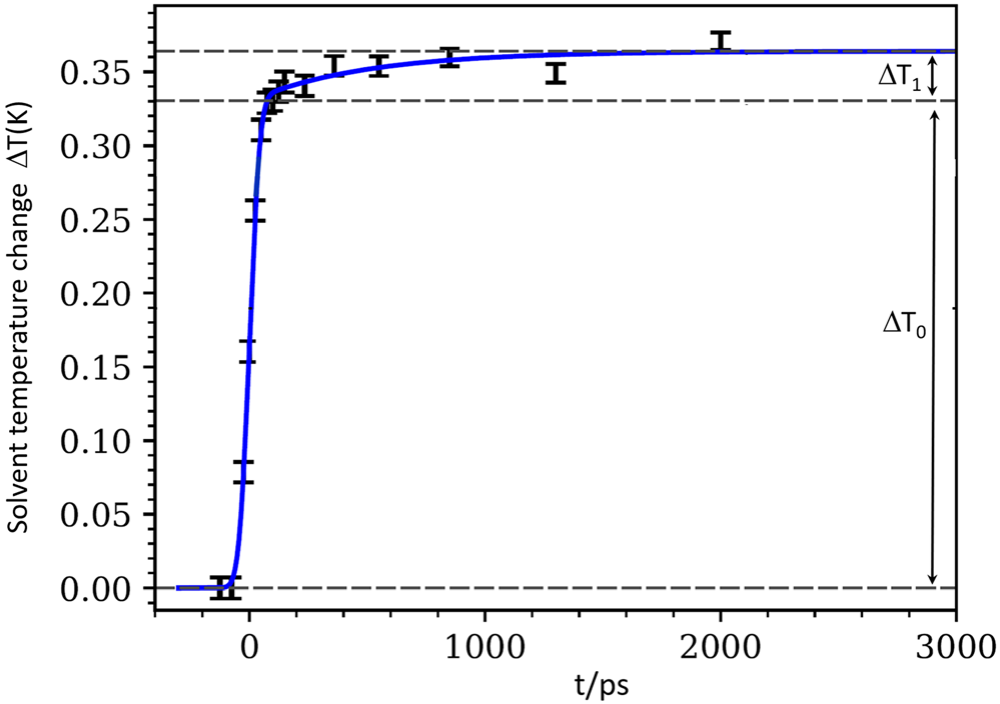
(a) Solvent temperature rise ΔT(t) during the photocycle. In the time range −50 to 50 ps, the steep rise ΔT_0_ is assigned to the ultrafast relaxation ^1^MLCT → ^3^MLCT → ^3^T_2_ → ^5^T_2_. These transitions are broadened by the 100 ps x-ray pulse. The slow rise from 50 ps to 2 ns is from the 725 ps transition ^5^T_2_(HS) → GS(LS). ΔT_0_ is a factor 2.1 greater than expected from the energy steps in the reaction.

The amplitudes of the solvent and solute/cage terms have to be consistent with the model of the reaction pathway, i.e. the rise in the solvent temperature has to match the change in energy of the photoproducts and their concentrations. The solvent temperature rise will now be examined. With the 100 ps solvent differential from the dye/water measurement, the temperature rise ΔT(t) is obtained from the equation

$${\Delta S}_{\text{Solvent}}(q,t)={\left(\frac{dS}{dT}\right)}_{|V}\times \Delta T(t),$$ (7)

and the result is shown in Fig. 8.

 The temperature curve is fitted to the expression

$$\Delta T(t)=\frac{1}{\sqrt{2\pi \sigma }}\;\int_{-\infty }^{\infty }dt\prime \;e^{-\frac{(t\prime -\tau {\;)}^{2}}{2\sigma^{2}}}\;\{\Delta T_{0}\;H(t\prime )+\Delta T_{1}\;H(t\prime )\;[1-e^{-\frac{t\prime }{\tau_{R}}}]\;\},$$ (8)

where H(t′) is the Heaviside step function, ΔT_0_ the transient rise from the ultrafast cascade to ^5^T_2_, ΔT_1_ the temperature increase from the depopulation of HS, *τ*_R_ the HS lifetime time, and *σ* is the length of the x-ray pulse. The Gaussian pulse length is *σ* = 42.5 ps for the 100 ps x-ray pulse (FWHM). The fit gives ΔT_0_ = 330 mK, ΔT_1_ = 34 mK, and ΔT_tot_ = 364 mK.

The slow rise ΔT_1_ = 34 mK is consistent with the expected rise from a 2.4 mM excited solution at time zero and that the ^5^T_2_ → GS decay deposits 0.62 eV of energy to the solvent. The excited state concentration derived from ΔT_0_, which was expected to deposit 2.48 eV (3.10–0.62) of energy per molecule, is 5.1 mM. If the photoexcitation was a single-excitation event in this experiment, the two concentrations should be equal. We will now examine the discrepancy in the temperature rise since this effect has been observed in other complexes as well.

## VI. SECONDARY EXCITATION OF HS

**FIG. 9.**
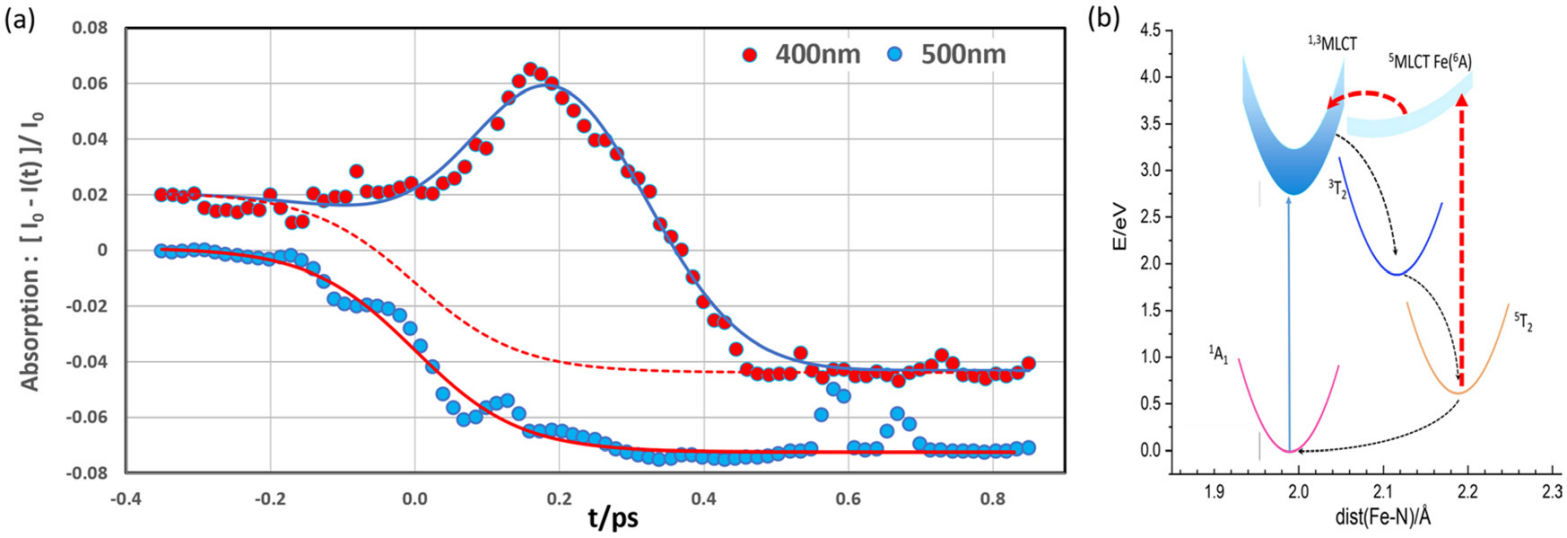
(a) Transient optical absorption of Fe^2+^(phen)_3_ in water after 400 nm excitation with a 150 fs pulse. (a) For the 500 nm probe pulse curve in blue, the decay around t = 0 is due to the depletion of the GS. For the 400 nm probe pulse curve in red, the peak at 0.19 ps is assigned to the ^5^T_2_^*^ → ^5^MLCT transition. (b) Energy curves showing the proposed ^5^T_2_^*^ excitation to the ^5^MLCT band followed by the transit to ^1,3^MLCT by ISC.

The excess solvent temperature rise might be explained by the fact that the 400 nm 1.2 ps pump pulse re-excites nacent ^5^T_2_^*^(HS) molecules to the ^5^MLCT band during the vibrational relaxation toward the bottom of the ^5^T_2_ potential. Note that the formation of the ^5^T_2_ state was indeed monitored by the ^5^T_2_ → ^5^MLCT transition at 340 nm (3.65 eV) in acetonitrile by Tribollet *et al.*^12^ Our 400 nm (3.10 eV) pump pulse might therefore excite ^5^T_2_^*^ into the ^5^MLCT band in the early stage of the vibrational relaxation process. Note that the excitation-during-relaxation technique was used by C. B. Harris *et al.* in their classical work that probed the vibrational relaxation of I_2_^*^ in CCl_4_ and in other solvents.^29^ The ^5^T_2_* → ^5^MLCT transition is spin allowed and the transient absorption should increase for a 400 nm (3.10 eV) pump pulse once the molecule has relaxed to match the 3.10 eV excitation window. The ^5^MLCT state might then transit to ^1^MLCT by ISC whereby the photocycle resumes. The re-excitation pathway is illustrated in Fig. 9(b).

To examine this hypothesis, an optical–optical experiment was performed in Mathias Bargheer's laboratory at the University of Potsdam. A 2 mM water solution of Fe^2+^(phen)_3_ was excited by 400 nm 150 fs pump pulses and probed with 150 fs pulses at 400 nm and 500 nm. The results are shown in Fig. 9(a).

When the solution is probed at 500 nm near the 512 nm absorption peak, the absorption decreases with time from the depletion of the GS. For the 400 nm probe however, a Gaussian-like peak with a FWHM of 0.36 ps appears 0.19 ps after the pump pulse. The peak is tentatively assigned to the transient absorption from the ^5^T_2_* → ^5^MLCT excitation with ^5^MLCT returning to ^1^MLCT by ISC. A second photocycle turn is thereby initiated. Double-excited molecules dissipate 2 × 3.1 eV to the solvent, triple-excited 3 × 3.1 eV and so on.

For the 400 nm probe, the amplitude of the depletion hole and the creation peak is −0.064 and +0.098, respectively, which means that the cross section *σ*(^5^T_2_* → ^5^MLCT) is 53% greater than *σ*(^1^A_1_ → ^1^MLCT). That shows the importance of the proposed re-excitation pathway.

## VII. SPIN STATES

While WAXS probes atom–atom distances, intra- and extra-molecular alike, WAXS is insensitive to the spin of the molecules. In contrast, XES is atom-specific and a powerful probe of the spin and valence states of transition metals. As shown below the XES signals from metal complexes in solution are feeble but essentially background free. XES is used here to show that the spin of Fe^2+^ is 0 and 2 in the LS and HS states respectively.

Upon the absorption of an x-ray photon at the K-edge of Fe^2+^, a 1s electron is ejected and leaving behind a 1s core hole. The core hole is filled by an outer shell electron accompanied by the emission of either an x-ray photon or an Auger electron. For the K-edge of Fe, the branching ratio is 34% x-ray emission and 66% electron emission.^30^ For the K_α_ and K_β_ lines, the emission is produced by the transitions 2p→1s and 3p→1s, respectively. The spin–orbit coupling of the 2p state splits K_α_ into two sub-lines K_α__2_ and K_α1_ from the transitions 2p_1/2_ (2e-) → 1s and 2p_3/2_ (4e-) → 1s with the parenthesis showing the electron occupancy in that state. The K_α2_ and K_α1_ lines peak at 6390.8 and 6403.8 eV, respectively. The energy splitting of the K_β_ emission from 3p_1/2_ (2e-) → 1s (K_β′_) and 3p_3/2_ (4e-) → 1s (K_β1,3_) is dominated by the exchange interaction between the 3p core hole and the 3d electrons. The spin–orbit interaction for Kβ is much weaker than for K_α_, about 0.7 eV (Ref. 31) due to the greater shielding of the nucleus. The central energy of K_β_ is 7058 eV for Fe.

XES is insensitive to the solvent hydrodynamics and the technique is ideal for monitoring population kinetics. One nice feature of XES compared with other spectroscopies is that the core holes can be produced by the pink beam which is ∼200 times stronger than the beam from a Si monochromator. The high beam intensity of 2 × 10^9^ ph/pulse from the undulator was essential in the present experiment due the low pump–probe frequency of 986.3 Hz.

The spin sensitivity of the K_β_ emission is due to the strong exchange interaction between the 3p core holes and the 3d electrons. The different spin states of Fe, singlet, doublet, …, quintet, have a unique K_β_ spectrum which is essentially independent of the ligand and the solvent.^32^ The valence-to-core (VtC) transitions 3d → 1s probe the ligand-modified 3d levels and VtC can therefore provide valuable information about the metal-to-ligand bonding.^33^ However VtC is two-orders of magnitude weaker than K_β_ and too weak to be used here due to the low pump–probe frequency (986.3 Hz).

**FIG. 10.**
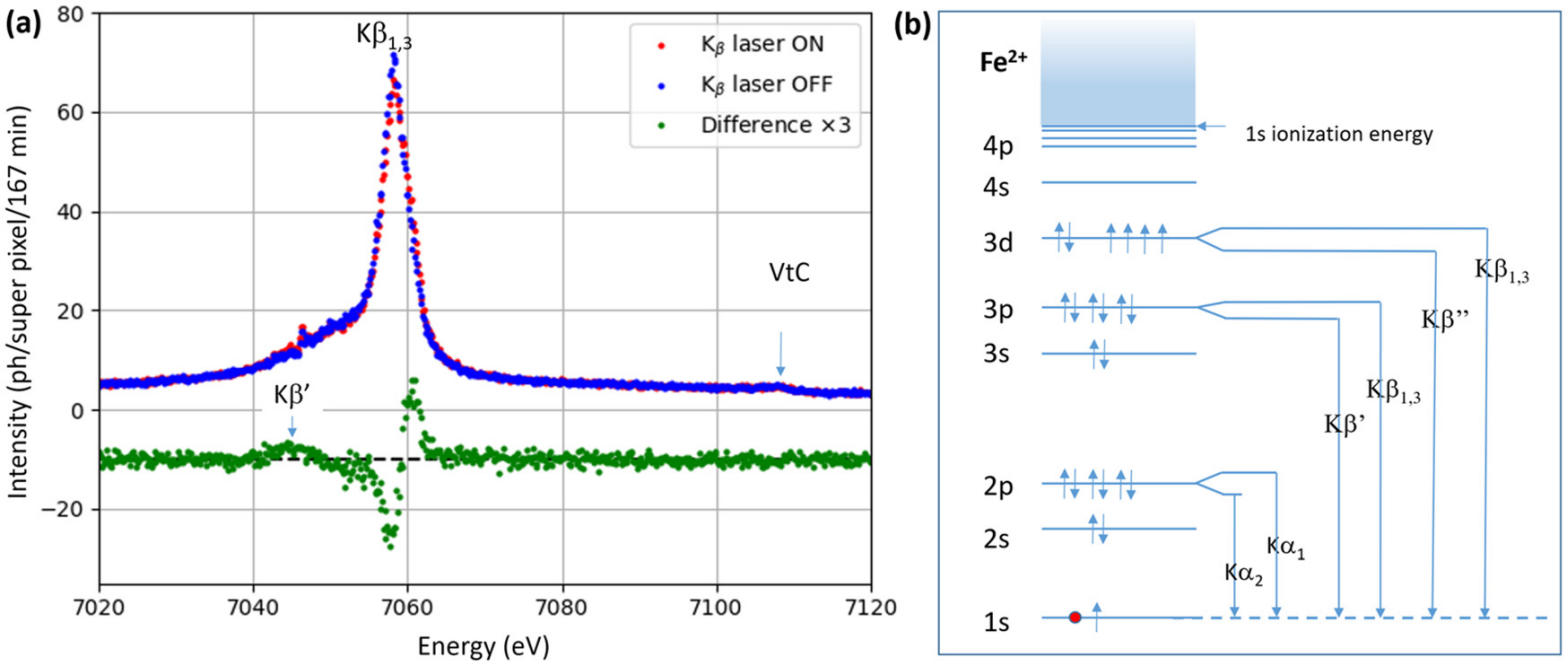
(a) K_β_ spectra for laser excited (ON, red) and non-excited (OFF, blue) Fe^2+^(phen)_3_ molecules. The ON data was recorded 100 ps after excitation. The difference spectrum ON–OFF is shown in green. (b) XES lines for Fe^2+^. The 3d spin configuration is shown in the exchange split HS state S = 2.

The K_β_ spectra ON and OFFand their HS-LS difference are shown in Fig. 10.

The shape of the difference spectrum HS(100 ps)−LS has the “pure” shape of the LS (S = 0) → HS (S = 2) transition since the non-excited molecules cancel out in the difference spectrum. The amplitude is proportional to the degree of excitation. The ON spectrum is shifted +0.2 eV from the OFF peak at 7058.3 eV. The appearance of the K_β_^′^ peak at 7044.6 eV is due to the increase in exchange splitting of the 3p level in the magnetic HS state. The VtC peak at 7110 eV is also detected; it is 100 times weaker than K_β_.

**FIG. 11.**
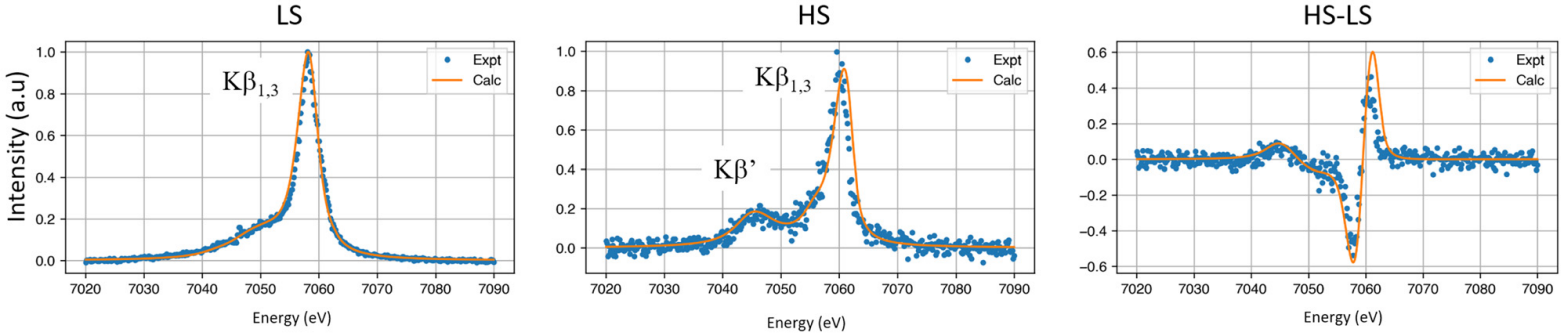
Measured and calculated spectra of the K_β_ emission from Fe^2+^(phen)_3_ in water. The curves in orange were calculated by CRISPY.

The K′_β_ and K_β1,3_ peaks in the HS spectrum are at 7045 and 7061 eV, respectively, corresponding to an exchange/spin–orbit splitting of 16 eV. The spectra in Fig. 11 were fitted to theoretical LS and HS spectra calculated by the CRISPY software. To match the experimental data, the calculated spectra were shifted +53 eV and broadened by 1.6 eV Lorentzians. The XES spectra from Fe^2+^(phen)_3_ in water resemble closely those for Fe^2+^(terpy)_2_ in water as reported by G. Vanko *et al.*^34^

## VIII. CONCLUSION

The photon-induced LS to HS transition of Fe^2+^(phen)_3_ in water was probed by TRWAXS and TRXES using synchrotron radiation with 100 ps resolution. The TRWAXS data, measured over the wide q range 0.5–10.7 Å^−1^, allowed to probe multiple length scales, from atom–atom distances inside the complex, the solvation structure around it, and the hydrodynamics of the solvent.

The difference scattering ΔS(q, t) is dominated by the change in the solvent temperature from the relaxation of the ^1^MLCT (< 1 ps) and HS (725 ps) states. After thermal correction of the scattering data, the HS and LS structures were compared with DFT/MD simulations that provided trial functions for the LS and HS states and their cage structures. The high-q range 4.0–10.7 Å^−1^ is in excellent agreement with the calculated Debye scattering from DFT with a 0.19 Å Fe–N bond elongation in the LS → HS transition. The elongation is caused by the antibonding character in the HS state (t_2g_)^4^(e_g_)^2^ that has two 3d electrons in the antibonding orbitals d_x2-y2_ and d_z2_.

The solvent cage around the LS and HS states were simulated by MD and the S_cage_(q) term was calculated from the g_i,j_(r) functions. MD predicts a 1.0 Å contraction of the first solvation shell and a positive cage amplitude in the low-q range 0–0.8 Å^−1^. The experiment shows that the cage amplitude is overestimated by MD and by reducing it to the 33% level, the fit to the data greatly improves. The amplitude reduction in q space corresponds to a 0.33 Å contraction of the cage radius in the HS state.

The time dependence of the solvent temperature was derived from the thermal solvent differentials from a separate dye/water experiment. The temperature rise from the slow 725 ps HS→LS relaxation is well explained by the energy difference of 0.61 eV between the HS and LS states and the initial HS concentration derived from the Debye fitting. In contrast, the temperature rise from the ultrafast (<1 ps) ^1^MLCT → HS transition is 2.1× greater than expected from the DFT calculated energy difference of 2.49 eV (3.10–0.61) and the HS concentration. The anomaly is caused by the re-excitation of nascent HS molecules by the 1.2 ps pump pulse at 400 nm. . Ultrafast optical/optical absorption measurements showed the appearance of a new absorption feature after 0.19 ps that is tentatively assigned to ^5^T_2_ → ^5^MLCT. ISC between ^5^MLCT and ^1^MLCT then provides a link that restarts the photocycle which explains the excess temperature rise of the solvent.

Finally, the spin states of LS and HS were probed by XES with the core-holes produced by a pink undulator beam. When the 100 ps difference spectrum is compared with reference spectra for putative spin changes, it is assigned to the S = 0 to S = 2 transition. In the HS state, the K_β1,3_ peak from the 3p_3/2_ → 1s transition has a weak side-peak K_β′_ from the transition 3p_1/2_ → 1s which is −16 eV from K_β1,3_ . The increase in the K_β′_ intensity is due to the greater exchange interaction in the magnetic HS state.

## Data Availability

The data that support the findings of this study are available within the supplementary material.
